# Synergistic Antitumor Effect between Gefitinib and Fractionated Irradiation in Anaplastic Oligodendrogliomas Cannot Be Predicted by the Egfr Signaling Activity

**DOI:** 10.1371/journal.pone.0068333

**Published:** 2013-07-18

**Authors:** Sophie Pinel, Jihane Mriouah, Marc Vandamme, Alicia Chateau, François Plénat, Eric Guérin, Luc Taillandier, Valérie Bernier-Chastagner, Jean-Louis Merlin, Pascal Chastagner

**Affiliations:** 1 Université de Lorraine, CRAN, UMR 7039, Campus Science, Vandoeuvre-les-Nancy, France; 2 CNRS, CRAN, UMR 7039, Vandoeuvre-les-Nancy, France; 3 Service d’Anatomie et Cytologie Pathologique, Hôpital de Brabois CHRU Nancy, Vandoeuvre-les-Nancy, France; 4 EA 4438, Université de Strasbourg, Strasbourg, France; 5 Service de Neurologie, Hôpital Central CHRU de Nancy, Nancy, France; 6 Institut de Cancérologie de Lorraine, Vandoeuvre-les-Nancy, France; 7 Service d’Onco-Hématologie Pédiatrique, Hôpital d’Enfants CHRU de Nancy, Vandoeuvre-les-Nancy, France; National Cancer Institute, United States of America

## Abstract

In high-grade gliomas, the identification of patients that could benefit from EGFR inhibitors remains a challenge, hindering the use of these agents. Using xenografts models, we evaluated the antitumor effect of the combined treatment “gefitinib + radiotherapy” and aimed to identify the profile of responsive tumors. Expression of phosphorylated proteins involved in the EGFR-dependent signaling pathways was analyzed in 10 glioma models. We focused on three models of anaplastic oligodendrogliomas (TCG2, TCG3 and TCG4) harboring high levels of phospho-EGFR, phospho-AKT and phospho-MEK1. They were treated with gefitinib (GEF 75 mg/kg/day x 5 days/week, for 2 weeks) and/or fractionated radiotherapy (RT: 5x2Gy/week for 2 weeks). Our results showed that GEF and/or RT induced significant tumor growth delays. However, only the TCG3 xenografts were highly responsive to the combination GEF+RT, with ∼50% of tumor cure. Phosphoproteins analysis five days after treatment onset demonstrated in TCG3 xenografts, but not in TCG2 model, that the EGFR-dependent pathways were inhibited after GEF treatment. Moreover, TCG3-bearing mice receiving GEF monotherapy exhibited a transient beneficial therapeutic response, rapidly followed by tumor regrowth, along with a major vascular remodeling. Taken together, our data evoked an “EGFR-addictive” behavior for TCG3 tumors. This study confirms that combination of gefitinib with fractionated irradiation could be a potent therapeutic strategy for anaplastic oligodendrogliomas harboring EGFR abnormalities but this treatment seems mainly beneficial for “EGFR-addictive” tumors. Unfortunately, neither the usual molecular markers (*EGFR* amplification, PTEN loss) nor the basal overexpression of phosphoproteins were useful to distinguish this responsive tumor. Evaluating the impact of TKIs on the EGFR-dependent pathways during the treatment might be more relevant, and requires further validation.

## Introduction

Gliomas are the most common form of primary brain tumor and correspond to a heterogeneous group of malignancies [1],[2], including the high-grade forms such as the anaplastic oligodendroglioma (AO), the anaplastic astrocytomas (AA) and the glioblastomas (GBM). Despite aggressive multimodal therapies, high-grade gliomas remain ultimately fatal: for example, the median survival for AO ranges between 3 and 10 years [3] while it does not exceed 15 months for GBM [4]. Consequently, extensive investigations are ongoing to improve current treatments and identify new molecular targets for therapy [5].

Abnormalities on the EGFR and the EGFR-dependent signaling pathways are the most frequently reported in high-grade gliomas and affect all histological classes [6]. They were associated with an unfavorable outcome [7],[8] and have been implicated in the development and aggressiveness of adult and paediatric high-grade gliomas [9]–[11]. EGFR signaling was shown to promote tumor cell proliferation and survival, invasion and angiogenesis [12]–[14] and mediate resistance to treatment, including ionizing radiation in preclinical models [15]–[17].

In this context, many clinical trials have evaluated EGFR tyrosine kinase inhibitors (gefitinib, erlotinib, lapatinib) in recurrent or progressive glioblastomas, or in newly diagnosed gliomas as a monotherapy or in addition to chemotherapy and/or radiotherapy [18]–[24]. Although clinical results were largely disappointing, small subsets of patients responded to TKIs-based treatments [22],[23],[25],[26]. Recently, a phase II study assessed the combination of gefitinib and irradiation in children newly diagnosed with a poor prognosis brainstem glioma: authors reported that three children (out of 43) experienced long-term progression-free survival (≥36 months), supporting the benefit of this combination in subgroups of patients [22].

The identification of these subsets of patients remains a challenge. In high-grade gliomas, determinants for EGFR tyrosine kinase inhibitor sensitivity, such as gene copy number, EGFR or EGFRvIII proteins expression, low phospho-Akt expression or PTEN loss have been investigated [25]–[28], overall with inconsistent results.

Preclinical experiments demonstrated that EGFR kinase inhibitors could radiosensitize glioma xenografts [29], without addressing the question about reliable biomarkers. Therefore, using experimental *in vivo* models, we investigated the radiosensitizing properties of gefitinib, attempting to identify the profile of responsive tumors.

## Materials and Methods

### Tumors

Each model was derived from a previously untreated high-grade glioma (according to the WHO classification and grading, 2007). Pieces of the patient tumor were subcutaneously transplanted into *nude* mice in the inguinal region near the femoral vessel, providing the first xenografts.

Each model was maintained *in vivo* by sequential passages in *nude* mice. Origin and molecular characterizations were summarized in Table 1 and Table S1.

**Table 1:** TCG2, TCG3 and TCG4 tumor characterization for oncogenic alterations commonly found in high-grade gliomas.

		Patient informations		Molecular Characteristics									
Tumor lines	Initial patient diagnosis/			EGFR and EGFR downstream signaling pathway						Other			
	Diagnosis after 2^nd^ reading^1^	Sexe	Patient age (years)	EGFR amplification*^2^*	EGFR variant III^3^	EGFR protein over expression^4^	Phospho-EGFR expression level^5^	PTEN expression^6^	PIK3CA mutations (exon 9 & 20)^7^	IDH1 mutation^8^	1p/19q co-deletion^9^	p53 status^10^	MGMT promoter methylation^11^
**TCG2**	AO	M	54	**+**	**–**	**yes**	679	–	WT	no	no	**MUT**	**M**
**TCG3**	AO	F	58	**+**	**–**	**yes**	2148	–	WT	no	no	**MUT**	**M**
**TCG4**	AO	M	72	**+**	**+**	**yes**	195	–	WT	no	no	WT	**M**

1 Primary diagnoses (determined in 2001 by a first pathologist according to the WHO classification 2000) were compared to a second opinion (given by an independent pathologist in 2013 according to the WHO classification 2007). AO  =  anaplastic oligodendroglioma.

*^2^*EGFR amplification was assessed using CGH array and FISH, leading to consistent results.

3 The expression of the EGFR variant III was determined by western-blotting.

4 EGFR protein overexpression was assesed by immunohistochemical detection and compared to non tumor tissue.

5 Phospho-EGFR expression level was determined using the Bio-plex phosphotrein arrays.

6 PTEN status was investigated using three techniques: CGH array, qRT-PCR and protein expression analysis by western-blotting, leading to consistent results. (-)  =  PTEN loss.

7 PIK3CA mutation analysis (exons 9 & 20) was performed using direct sequencing.

8 IDH1 mutation was assessed by immunohistochemistry;

9 1p/19q Codeletion was determined using microsatellite analysis for loss of heterozygosity on chromosome 1 and 19q, as previously described. In parallel, IHC studies showed no expression of alpha-internexin.

10 p53 status was determined by FASAY and confirmed by IHC: WT  =  wild type or MUT  =  Mutated.

11 MGMT promotor methylation status was evaluated with the methylation specific polymerase chain reaction after DNA modification by sodium bisulfite: M  =  mutated or U  =  unmethylated.

### Animals

Pathogen-free, 5–7 week-old female athymic NMRI-nu *(nu/nu)* mice were purchased from Janvier Laboratories (Le-Genest-St-Isle, France). Animals were housed in solid-bottomed plastic cages (6 mice per cage) with free access to tap water and food *ad libidum*. All experiments were performed in accordance with animal care guidelines (Directive 2010/63/EU) and carried out by competent and authorized persons (personal authorization number 54–89 issued by the Department of Vetenary Services) in a registered establishment (establishment number C-54-547-03 issued by the Department of Vetenary Services). Tumor grafts, radiotherapy and resection were performed under general anesthesia as previously described [30]. At the end of experiments, mice were euthanized with anesthetic overdose (pentobarbital injection).

### Treatment procedures

Mice were randomly assigned into four groups (Fig S1): control (CTRL), gefitinib (GEF), radiotherapy (RT) and gefitinib + radiotherapy (GEF+RT). In the CTRL group, mice were injected with saline. GEF (AstraZeneca Ltd, UK) was administered *i.p.* at a daily dose of 75 mg/kg. In the RT group, mice received 5 fractions of 2 Gy per week, as previously described [30]. In the GEF+RT group, they received the combination of GEF and RT, with GEF given 4h before irradiation. Treatments started when tumor volume reached V_0_  = 250+/−50 mm^3^ and were delivered for 2 weeks. For morphological and biological analysis, tumors were excised 24 h after the last treatment administration at the end of the first (Day 6) or second week (Day 13).

### Antitumor effect of treatments

Tumor volume was determined three times per week, measuring two perpendicular diameters with a calliper. Animal were sacrificed when the tumors reached five times their initial volume (5V_0_), thus defining the ‘survival times’. Tumor volumes, tumor growth delays (TGD), and the enhancement *ratio* (ER) were calculated as previously described [30],[31]. Complete responses were defined as the complete disappearance of a measurable tumor mass at some point after initiating therapy and maintained for at least 120 days.

### Detection of VEGF in tumor

Whole cell protein extracts were prepared from frozen tumor tissues *(Nuclear extract kit, Active Motif, Belgium) (Methods S1)*. VEGF concentrations in tumor lysates were determined by ELISA (Quantikine ELISA kit, R&D System, France).

### Phosphoproteins expression analysis

The expression of phospho-EGFR, phospho-AKT and phospho-MEK1 were analyzed using Bio-Plex® phosphoprotein array (Bio-Plex®, Marnes-la-Coquette, France). BPA assay was performed on whole cell protein extract as previously reported [32],[33]. This technique based on multiplex sandwich bead immunoassays is detailed in *Methods S1*. The expression level of each phosphoprotein was given as relative fluorescence intensity in an arbitrary unit.

### Morphological analysis

5 µm-thick sections of formalin-fixed paraffin-embedded tumor tissue were evaluated using conventional histology and immunohistochemistry methods for determination of the proliferation and apoptotic indexes through the detection of Ki67 antigen and cleaved caspase-3 protein, respectively [34]. Methods are mentioned in *Methods S1*.

Immunohistochemical detection of CD31 and type IV collagen was carried out on 5 µm-thick adjacent sections of tumor samples fixed in a zinc fixative (Tris-HCl 0.1M pH 7.4, 3.2 mM calcium acetate, 22.8 mM zinc acetate, 36.7 mM zinc chloride). All antibodies were diluted in a 0.1 M PBS, 0.3% (^m^/_v_) BSA, 0.1% (^m^/_v_) sodium azide, 0.06% (^m^/_v_) n-ethylmaleimide and 20% (^v^/_v_) glycerol (PAB) buffer. The sections were incubated overnight at 4°C with either a primary rabbit anti-mouse collagen IV antibody (diluted 1/3000, Novotec, Wittelsheim, France) or a rat anti-mouse CD31 monoclonal antibody (clone MEC13.3, diluted 1/400, BD Pharmingen, Le Pont de Claix, France). Then, the sections were washed in two changes of PBST (0.1 M phosphate buffer, pH 7.4, 0.1% (^v^/_v_) Tween 20) over 10 min and incubated for one hour at room temperature with a biotin-conjugated secondary antibody (a goat anti-rabbit antibody, diluted 1/200, Dako, Trappes, France or a rabbit anti-rat antiserum, diluted 1/400, Chemicon, Lyon, France). After two additional 10 min washes, secondary antibodies were detected with a streptavidin-488 conjugate (Streptavidin fluoroprobe 488, Interchim, Monluçon, France, diluted 1/4000), in the case of CD31 or a Streptavidin-Texas Red conjugate (Streptavidin – SR 101 – diluted 1/8000, Interchim, Montluçon, France) in the case of collagen IV. Image acquisition was carried out using a fluorescent microscope (Axiophot II; Zeiss, Jena, Germany) equipped with a cooled AxioCam HRc CCD camera (Zeiss) controlled by the Axiovision 4.4 digital image processing software.

### Statistical analysis

The Mann-Whitney *U* test was used to evaluate the statistical significance of the results. Kaplan-Meier curve analysis was performed using the Log-rank test. Statistical analysis was performed using GraphPad 5.0 software (GraphPad Prism 5.0 Software). Differences were considered significant at *p* values <0.05.

## Results

### EGFR and EGFR-dependent pathways activation in glioma xenograft models

The first step consisted in validating the experimental use of our glioma xenograft models (4 derived from AO and 6 derived from GBM). In gliomas, different types of EGFR aberrations were described – *EGFR* gene amplification, multiple exon deletion (EGFR variant III), autocrine loop and overexpression – and all of them result in the activation of the receptor. Hence, we sought to describe the EGFR activation in our 10 tumor lines, studying the expression of phosphorylated EGFR (phospho-EGFR) and downstream signaling proteins (phospho-AKT and phospho-MEK1). To do that, we performed a multiplex immunoanalysis using the Bio-Plex® phosphoprotein array.

**Figure 1:**
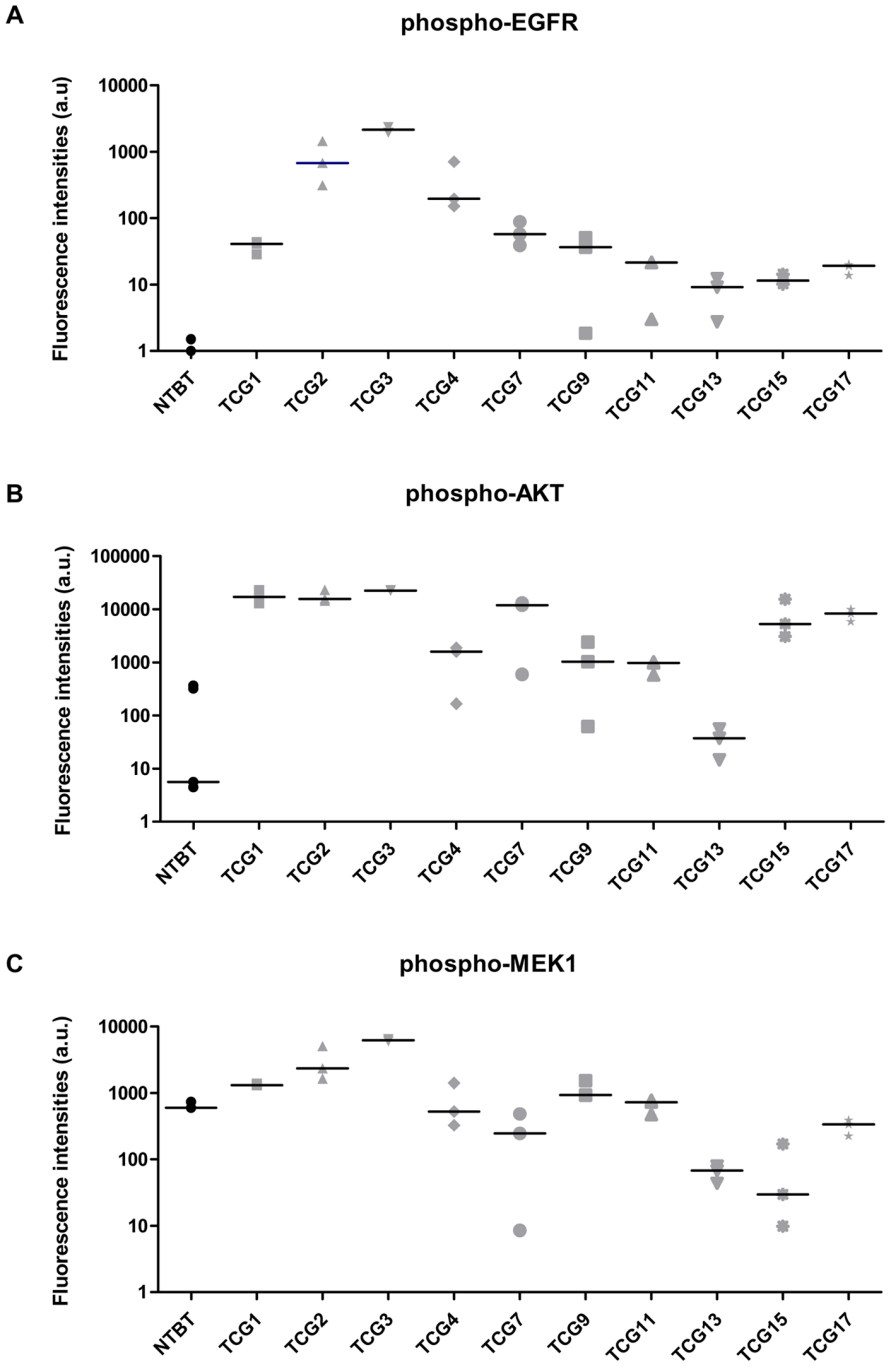
EGFR and downstream signaling phosphorylated proteins expression in 10 human malignant glioma xenograft models. Expression of (**A**) phosphorylated EGFR (phospho-EGFR) and downstream signaling proteins: (**B**) phospho-AKT and (**C**) phospho-MEK1 were measured by BPA assay. For each model, fluorescence intensity values corresponding to 3 independent tumors were plotted and the median was represented by the bar. *NTBT: non-tumor brain tissue; a.u.  =  arbitrary units.*

As shown in Figure 1A, whatever the tumor line, phospho-EGFR was always significantly overexpressed as compared to the non-tumor brain tissue. However, large variations in expression levels were observed between the tumor lines and no relationship was found between the expression level of phospho-EGFR and the histological subtype (AO or GBM). In all samples, phospho-EGFR overexpression was associated with a clear overexpression of phospho-AKT (Fig. 1B). In contrast, only 50% of glioma xenograft models showed a phospho-MEK1 overexpression, as compared to the non-tumor brain tissue, and, when overexpressed, phospho-MEK1 expression level did not exceed a 10-fold increase (Fig. 1C). Figure 1 also shows that phospho-EGFR levels do not correlate with phospho-Akt or phospho-MEK1 as tumors with low phospho-EGFR (TCG1, TCG17) have also high phospho-Akt and phospho-MEK1. This is a well-known phenomenon as gliomas often harbor other mutations which can also up regulate phospho-Akt and phospho-MEK1.

Further oncogenic alterations commonly found in high-grade gliomas were also analyzed in our xenograft models (Table 1 and Table S1, Fig. S2). In particular, we focused on three models of anaplastic oligodendrogliomas (TCG2, TCG3 and TCG4) that harbored the highest phospho-EGFR expression levels. *EGFR* amplification and PTEN loss were found in these models and neither 1p/19q co-deletions nor IDH1 mutations were detected.

### Difference in treatment response profiles

**Figure 2:**
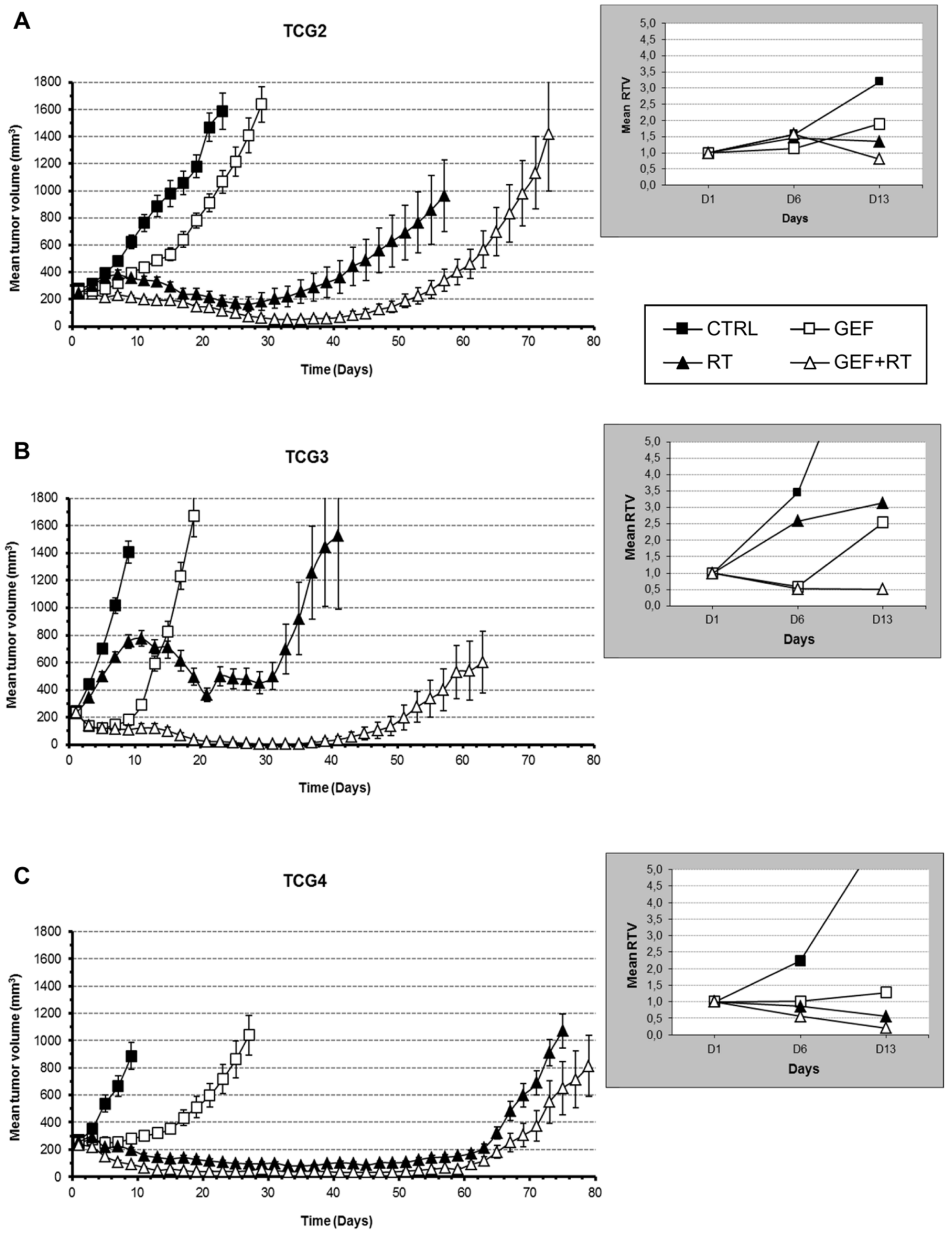
Effect of treatments on (A) TCG2 (B) TCG3 and (C) TCG4 tumor growth. Xenografts-bearing mice were randomly assigned into four therapeutic groups (6–14 mice per group): CTRL (▪), GEF (□), RT (▴) and GEF+RT (▵). Treatments started at D1 and were administered for two consecutive weeks. Results are expressed as the mean tumor volume (± SEM) evolution. In each xenograft model, inset focuses on the mean relative tumor volume (mean RTV) on days 6 (D6) and 13 (D13), as compared to day 1 (D1).

Because they presented the highest phospho-EGFR expression levels, along with high expression levels for phospho-AKT and phospho-MEK1, TCG2, TCG3 and TCG4 tumor lines were expected to benefit from gefitinib-based treatment. Hence, they were used to evaluate the radiosensitizing properties of gefitinib (Fig. 2). Despite high similarities in their genetic profiles, the response profiles of these three models were very different.

#### GEF treatment induced a biphasic response in TCG3 xenografts

In the three models studied, GEF induced slight but significant tumor growth delays reaching 4.5 (*vs* CTRL, p = 0.0042), 8.5 (*vs* CTRL, p = 0.0001) and 16 days (*vs* CTRL, p = 0.0001) for TCG2, TCG3 and TCG4, respectively (Table 2). However, major differences were noticed in tumor growths during the treatment (D1 to D13).

**Table 2:** Tumor growth delays (TGD) and Enhancement *ratios* (ER).

	TCG2 model			TCG3 model			TCG4 model		
	Complete responses	Median survival (days)	TGD (days)	Complete responses	Median survival (days)	TGD (days)	Complete responses	Median survival (days)	TGD (days)
**CTRL**	0/9	20	_	0/14	8	_	0/11	12	_
**GEF**	0/12	24.5^a^	4.5	0/14	16.5^a^	8.5	0/8	28^a^	16
**RT**	0/7	62^a^	42	0/12	37^a^	29	0/11	77^a^	65
**GEF+RT**	0/13	80^a, b^	60	6/13 (46.1%)	118^a, b^	100	0/6	84.5^a, b^	72.5
***ER***			***1.29***			***2.67***			***0.90***

Complete responses were defined as the complete disappearance of a measurable tumor mass at some point after initiating therapy and maintained for 4 months. Median survival (in days) corresponds to the time needed by the tumors to reach five times their initial volume. TGD corresponds to the difference (in days) between median survival in treated group and median survival in CTRL group. ER was defined as TGD_GEF+RT_ / [TGD_GEF_ + TGD_RT_]. ^a^Comparison *vs* CTRL, p<0.05; ^b^Comparison *vs* RT, p<0.05.

In TCG2 and TCG4 xenografts, GEF injections induced tumor volume stabilization that *in fine* led into the right shift in the tumor growth curves (Fig. 2A and 2C), as compared to CTRL groups. So, mean RTV for the TCG2 xenografts were 1.1 and 1.9 on Day 6 and Day 13, respectively (Fig. 2A inset). By contrast, we noticed in the TCG3 model an unexpected biphasic response between D1 and D13. GEF firstly induced a dramatic shrinkage of the tumor mass, which was followed by a rapid regrowth in spite of continued treatment (Fig 2B inset). Mean relative tumor volume (RTV) dropped to 0.5 on Day 6 (*i.e.* end of the 1^st^ week of treatment) and rapidly increased up to 2.5 on Day 13 (*i.e.* end of the 2^nd^ week of treatment). TCG3 tumor line behavior clearly evokes a therapeutic escape after a first phase of a high responsiveness to GEF.

#### Tumor response to irradiation

Fractionated radiotherapy alone induced significant tumor growth delay whatever the model considered (Table 2, Fig. 2 and S3). As expected, the RT-induced tumor regression was delayed, beginning several days after treatment onset (D8 for TCG2 and D10 for TCG3) and lasting for few weeks. *In fine*, RT-treated xenografts systematically regrew.

In the 3 tumor lines, the combined treatment GEF+RT was significantly more effective than the monotherapies (GEF or RT). However, TCG3 xenografts behavior was particularly remarkable. First, TCG3 model was 1.4-fold and 2.2-fold more radioresistant than TCG2 and TCG4, respectively and strongly benefited from the combined treatment GEF+RT. Actually, GEF+RT allowed to triple the median survival of TCG3 xenograft-bearing mice (118 days for GEF+RT *vs* 37 days for RT, p<.0001) while the median survival in TCG2 was only increased from 62 to 80 days (p = 0.0017) (Table 2). Moreover, in the TCG3 model, a complete response was obtained in 6 of 13 tumors when treated by GEF+RT. Enhancement *ratios* (ER) reaching 2.67 in TCG3 tumor line indicates a strong synergistic interaction between GEF and RT. In contrast, only additive interaction or worst, infra-additive interaction were noticed in the TCG2 (ER  = 1.29) and TCG4 xenografts (ER  = 0.90), respectively (Table 2).

### GEF induced strong inhibition of EGFR-dependent signaling pathways in TCG3 xenografts, but not in TCG2 tumors

The effect of treatments on activation of EGFR-downstream signaling pathways was assessed in TCG2 and TCG3 models. Tumors were harvested at mid treatment (Day 6).

**Figure 3:**
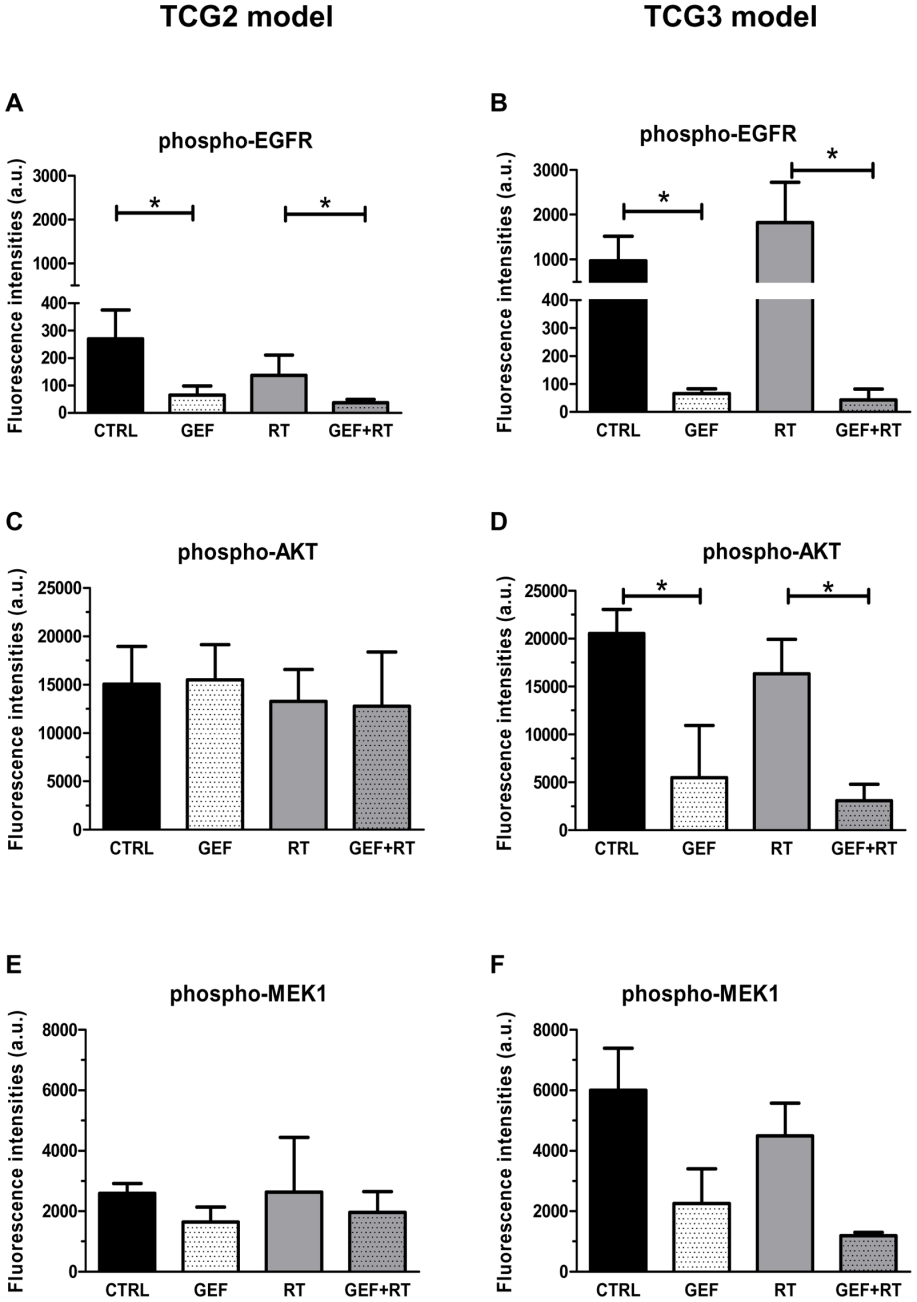
Effect of gefitinib and/or fractionated radiotherapy on phospho-EGFR and downstream phosphoproteins. On D6, phospho-EGFR (**A, B**), phospho-AKT (**C, D**) and phospho-MEK1 (**E, F**) expression were assessed in TCG2 (**A, C, E**) and TCG3 (**B, D, F**) xenograft-bearing mice which received saline (CTRL), GEF, RT or GEF+RT treatments for one week. Expression of phosphoproteins is presented as fluorescence intensity (mean ± SD) measured by BPA assay (a.u.  =  arbitrary units) (n = 6 independent tumors), *p<0.05.

In both models and whatever the phosphoprotein considered, no significant alteration was observed with RT alone (Fig. 3). In contrast, phospho-EGFR expression was significantly decreased in TCG2 and TCG3 xenografts when mice received GEF, confirming that the EGFR inhibitor reached its target (Fig. 3A and 3B). GEF activity was mainly marked in TCG3 tumors since a near complete abrogation of EGFR phosphorylation was observed in GEF and GEF+RT groups (Fig. 3B).

Results concerning downstream signaling through PI3K/AKT (Fig. 3C and 3D) and MEK/ERK (Fig. 3E and 3F) pathways differed between TCG2 and TCG3 models. In TCG2 xenografts, GEF alone or its combination with RT caused no change in phospho-AKT and phospho-MEK1 expression (Fig. 3C and 3E). In contrast, AKT phosphorylation in the TCG3 tumors was 4- to 5-fold reduced in tumors exposed to GEF or GEF+RT (GEF *vs* CTRL, p = 0.0022; GEF+RT *vs* RT p = 0.0022) (Fig. 3D). Even though not significant, similar reduction of MEK1 phosphorylation was observed when mice received GEF alone or in combination with RT (Fig. 3F).

### Treatments induced major morphological changes in TCG3 xenografts

**Figure 4:**
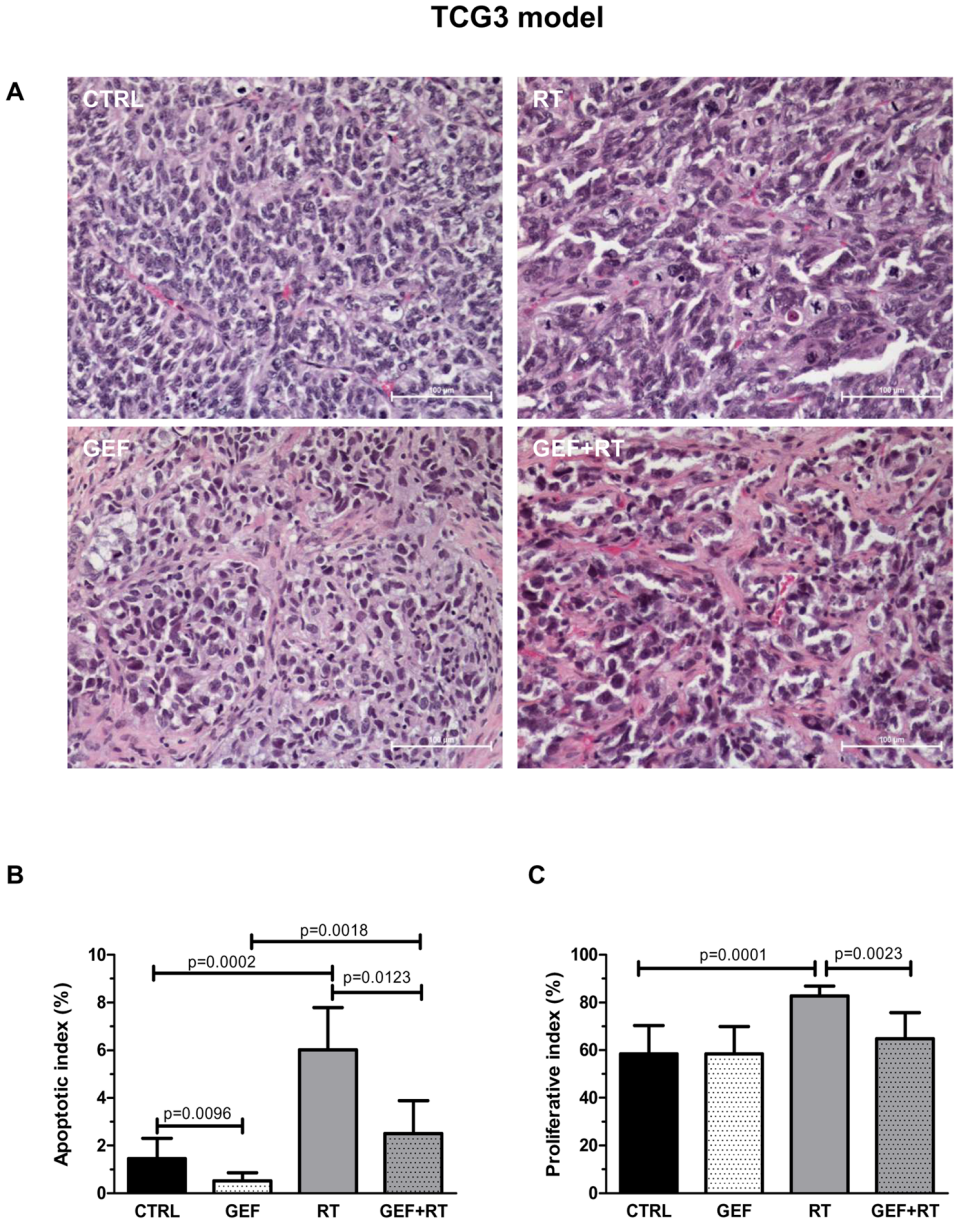
Gefitinib and/or fractionated radiotherapy induced morphological changes in TCG3 glioma xenografts. Tumors were harvested on D6, 24h after the last treatment fraction. Representative micrographs (**A**) of TCG3 xenograft sections after saline (CTRL), GEF, RT or GEF+RT treatment (HES staining). (**B**) The apoptotic index corresponds to the percentage of positively labeled cells for cleaved caspase-3. (**C**) The proliferative index corresponds to the percentage of tumor cells positively labeled for Ki-67. In order to determine proliferative and apoptotic indexes, a minimum of 1,000 cells were counted for each tumor. Results are expressed as the mean ± SD of at least four tumors.

To further elucidate the mechanisms involved in the interaction between gefitinib and ionizing radiation, immunohistochemical analysis were performed in TCG2 and TCG3 tumors harvested from mice killed at mid treatment (Day 6) (Fig. 4).

In the CTRL groups, TCG2 and TCG3 tumors exhibited high cellular density and high proliferative activity, with few atypical mitosis. TCG2 tumors showed more extensive necrosis than TCG3 (Fig. S4A).

In both models, histological examination of tumors exposed to fractionated irradiation showed accumulated aberrant mitotic figures (hyperploidy, formation of giant cells with abnormal nuclei), characteristic of RT-induced mitotic catastrophe (Fig. 4A and S4B).

Histological responses to the GEF showed marked differences between TCG2 and TCG3 models. In TCG2 tumors, low alterations were observed as compared to CTRL group, except a lower cellular density. In the TCG3 model, in contrast, histological examination of tumors that had regressed after GEF treatment showed islets of remaining tumor cells, with an increase of the extracellular matrix (Fig. 4A and S4B).

In both models, in the GEF+RT groups, morphological features resulted from the addition of effects induced by each treatment. Hence, in the TCG3 xenografts, we mainly observed islets of giant cells with abnormal nuclei separated from each other by large spans of fibrotic extracellular matrix.

In TCG3 tumors, the effects of treatments on cell death were assessed through the apoptotic index determination after immunochemical detection of the cleaved caspase-3 (Fig. 4B). Significant decreases in the apoptotic index on Day 6 were noticed in the GEF and GEF+RT groups, as compared to the CTRL and RT groups, respectively. Taking into account that the cellular density also decreased in these treatment groups, this may indicate that a massive apoptotic response has been rapidly triggered after GEF treatment onset, leading to apoptotic cells loss before tumor excision.

Next, we investigated the effects of treatments on cell proliferation through the immunochemical detection of the Ki-67 antigen. The proliferative index was significantly increased in TCG2 and TCG3 irradiated tumors (Fig. S4C and 4C), illustrating a RT-induced G2 arrest. Given that the proliferative index was lower in GEF+RT group than in RT group (64.8+/−11.0 *vs* 82.8+/−4.2; p = 0.0023), these results suggest that GEF was able to attenuate the RT-induced G2 arrest, consistently with recent data in lung cancer cells [35]. In TCG2 xenografts, the proliferative index was significantly reduced when mice received GEF alone as compared to CTRL (44.9+/−6.8 *vs* 58.0+/−3.4; p = 0.0159) (Fig. S4).

### GEF induced a vasculature remodeling in TCG3, but not in TCG2 tumors

**Figure 5:**
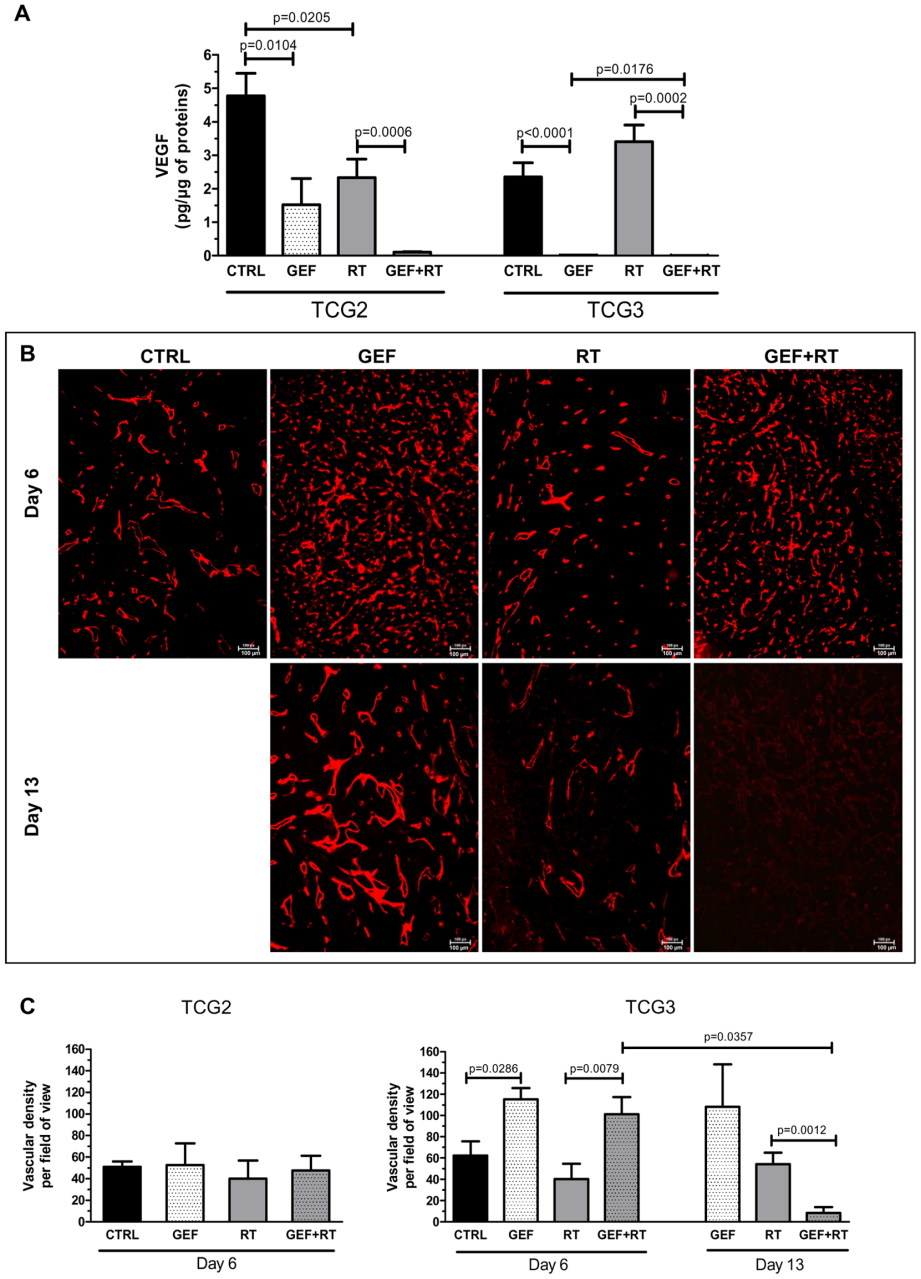
Impact of treatments on VEGF concentration and tumor vasculature in TCG2 and TCG3 models. Tumors were harvested on days 6 (D6) and 13 (D13), 24 h after the last fraction of saline (CTRL), GEF, RT or GEF+RT treatment. (**A**) VEGF concentrations measured in tumors by ELISA assay (Day 6). (**B**) Immunohistochemical detection of the basement membrane of tumor blood vessels based on mouse type IV collagen staining in TCG3 xenografts. (**C**) The vascular density corresponds to the number of CD31 and type IV collagen positive vessels counted in a field of view (X 200 magnification) for at least four tumors. Results are expressed as the mean ± SD.

Inhibition of EGFR has been shown to induce tumor vascular changes known as “vascular normalization” through an indirect decrease of VEGF production, and this was shown to improve chemotherapy or radiotherapy efficacy [36],[37]. We thus investigated the impact of the different treatments on tumor VEGF concentration and tumor vasculature. Figure 5A shows that in both glioma models *in vivo,* GEF alone or combined with RT induced a spectacular decrease in VEGF level. The effect was higher in TCG3 xenografts receiving GEF+RT in which VEGF level was 400-fold lower than in the CTRL group.

To assess the influence of treatments on tumor angiogenesis, tumor sections were immunostained for CD31 and Collagen IV (Fig. 5B, S4 and S5). The staining patterns of both antibodies superimposed strictly (Fig. S5 and S6A).

In TCG2 xenografts, tumor vasculature was not modified (Fig. S6B) and vessel density was similar whatever the treatment delivered, ranging from 40 to 50 CD31-positive vessels per field of view (Fig. 5C).

In TCG3 model in contrast, tumor vasculature exhibited dramatic quantitative and qualitative changes (Fig. 5B, 5C and S5). On Day 6, whereas ionizing radiations alone had no significant effect on tumor vessel density (40.2+/−14.4 *vs* 62.5+/−13.3 in the CTRL group), tumors in mice treated with GEF alone had significantly higher CD31-positive vessels (115.3+/−10.5; p = 0.0286) than CTRL, representing approximately a two-fold increase (Fig. 5C). On Day 6, vessels in the GEF group, although more abundant, exhibited very thin lumen as compared to enlarged vessels that were observed in the CTRL and RT groups (Fig. 5B). On Day 13, tumors in GEF group exhibited as many vessels as on Day 6, but their lumen became obviously larger. In GEF+RT group, vessel density was first increased (Day 6: 101.2+/−16.1; *vs* RT p = 0.0079) then, significantly reduced as it dropped to less than 10 vessels per field of view (Day 13: 8.3+/−5.5). Vessel lumens in the GEF+RT group collapsed.

## Discussion

Because they displayed the highest EGFR signaling activation, three tumor lines (TCG2, TCG3, and TCG4) were selected to evaluate the antitumor effect of gefitinib combined with irradiation, as compared to GEF alone and ionizing radiation alone. These three models derived from anaplastic oligodendrogliomas but did not harbor the typical alterations, namely the 1p/19q co-deletion and the IDH1 mutation. In contrast, molecular abnormalities in these xenograft models affected the EGFR (*EGFR* gene amplification, EGFR overexpression, EGFR variant III) associated with PTEN loss. Recent works attempted to identify molecular subtypes in glioblastomas and diffuse gliomas [7],[38],[39]. Genetic profiling defined four subtypes in GBM (proneural, neural, classic and mesenchymal) and three of them (proneural, classic and mesenchymal) were similarly described in AO [7]. Even if the “classic profile” was not prevalent (∼15%) in AO, as compared to the proneural one (∼75%), this subclass allowed to distinguish oligodendroglial tumors with neither 1p/19q co-deletion nor IDH1 mutations but with *EGFR* gene amplification and chromosome 10 loss. More important, Cooper *et* *al.* reported a significantly worse outcome for the “classic subclass” of AO with a median survival <20 months (*vs* >60 months for proneural subtype) and 2 years-survival rate of about 40% (*vs* >80% for proneural), highlighting the need of new therapeutic strategies for these tumors. Molecular profiles of TCG2, TCG3 and TCG4 tumors were concordant with the “classic subtype” of AO and the high phospho-EGFR level found in these models has logically argued for their use to evaluate the combined treatment GEF+RT.

Our results confirmed that this therapeutic combination could be efficient to treat anaplastic oligdendrogliomas and that gefitinib could act as a great radiosensitizer, as previously shown for other tumor models [29],[35],[40],[41]. However, only one tumor line (out of 3) was highly responsive to the combined therapy. Actually, in TCG3 xenograft model, the enhancement *ratios* (ER) that reached 2.67 pointed out a great synergistic interaction between GEF and RT, leading to tumor cure in ∼50% of cases. In contrast, only additive or infra-additive interaction were noticed in the TCG2 (ER  = 1.29) and TCG4 xenografts (ER  = 0.90). Our preclinical data are consistent with clinical trials in which only a small subset of patients benefit from the TKIs-based treatments [22],[42].

All selected tumor lines (TCG2, TCG3, and TCG4) were characterized by high basal phospho-EGFR expression, along with phospho-AKT and phospho-MEK overexpression, supporting the activation of the EGFR signaling pathway in tumors. As gefitinib-mediated radiosensitization was restricted to only one model, this clearly demonstrates that basal phospho-EGFR, phospho-AKT and phospho-MEK overexpressions cannot be considered as reliable markers to predict treatment synergy between anti-EGFR agent and irradiation. Similarly, classical molecular markers such as *EGFR* gene amplification and PTEN loss were not useful to predict treatment efficacy. Especially, PTEN loss did not preclude GEF-mediated radiosensitization in TCG3. This result contrasts with previous studies reporting a significant role of PTEN loss in TKIs treatment failure [13],[43].

Further investigations were thus performed to characterize the profile of responsive tumors when treated by combined therapy GEF+RT. Even though most of oncogenic alterations were similar between TCG2 and TCG3, behaviors of these tumor lines were particularly different, prompting us to focus biological and morphological analysis on these models.

GEF (with or without RT) was shown to induce, five days after the treatment onset, a critical inhibition of EGFR-dependent signaling pathways in TCG3, but not in TCG2 xenografts. The lack of pathways blockade in TCG2 model despite the EGFR dephosphorylation corroborates recent clinical data demonstrating that gefitinib has reached the tumor, efficiently dephosphorylated the target, but was not able to control the pathways activity [28].

While GEF monotherapy showed modest activity leading to a slight but significant tumor growth delay in TCG2 model, it induced a dramatic shrinkage of the TCG3 tumor mass rapidly followed by a tumor regrowth, typical of a therapeutic escape. Such a tumor escape from GEF control was hypothesized to be typical of an acquisition of drug resistance. In non-small lung cancer, a missense mutation (*T790M)* within the EGFR kinase domain has been shown to emerge in lesions that progress while on TKIs, yielding a protein with reduced binding to drug [44]. However, this mutation was not found in TCG3 xenografts, ruling out this hypothesis to explain the treatment failure in our model *(data not shown)*. More likely, the therapeutic escape after GEF treatment in TCG3 tumors may be associated with the important vascular remodeling including an increase in vascular density and vessels lumen diameters despite a dramatic decrease in VEGF levels. Similar observations have been previously reported for tumors that recurred during chronic suppression of angiogenesis with anti-VEGF [45]: the remodeled vasculature potentially supports increased perfusion and recurrent tumor growth. In contrast, the combined treatment GEF+RT resulted in vascular destruction *in fine*, preventing vascular remodeling and recurrence. This mechanism can explain the unexpected high tumor cure rate (∼50%) observed in TCG3 models as Calabrese *et* *al.* demonstrated that depletion of blood vessels from xenografts ablated cancer stem cells (CSC) from tumors, impairing tumor regrowth and recurrence [46].

We are aware that subcutaneous tumor xenografts do not recapitulate the invasive growth patterns of patient oligodendrogliomas and tumor vasculature differs between heterotopic and orthotopic tumors. Furthermore, the blood-brain-barrier naturally interferes with the drug delivery. Hence, our promising and informative results have to be confirmed in orthotopic models that are clinically more relevant animal models.

Taken together, our results highlight that TCG3 xenografts displayed an “EGFR-addictive” behavior which favored the radiosensitization by GEF. Indeed, the concept of “oncogene addiction” corresponds to the mechanism by which a tumor becomes largely dependent on a single activated oncogene for proliferation, but above all, for survival [47],[48]. Sudden inhibition of this dominant oncogenic signal results in a rapid and important cell death that can be rapidly compensated by the emergence of resistance due to other oncogenes activation [48],[49]. Consistently, the combination of TKI with ionizing radiation in this context of “EGFR-addictive” tumors prevented the occurrence of resistance and was a relevant therapeutic strategy to treat AO xenografts.

Unfortunately, this study demonstrates that the basal overexpression of phospho-EGFR, along with the overexpression of phospho-AKT and phospho-MEK1, surrogate markers of the EGFR- pathways activation in tumors, are not sufficient to predict this “EGFR-addiction” in oligodendrogliomas. By contrast, biological and vascular responses of tumors to TKIs measured in the first days post-treatment (D6) that obviously differed between TCG2 and TCG3 models seem indicative to distinguish the EGFR-addictive tumors from others and then, to identify responsive tumors to GEF+RT. Similarly, radiosensitization by lapatinib, another small tyrosine kinase inhibitor targeting both EGFR and HER2, was shown to correlate with inhibition of phosphorylated AKT in breast tumor xenografts [50]. Hence, measuring the impact of TKI treatment on EGFR-signaling pathways could be useful and more relevant than determining the basal EGFR activity to predict long-term efficacy of combined therapy GEF+RT. This implies that TKI administration occur few days before tumor removal. Such an approach is clinically feasible as reported in two recent phase II trial [28],[42]: herein, recurrent gliomas were treated for 5 to 7 days with gefitinib or erlotinib before surgery and resected tissues were evaluated for EGFR-dependent phosphoproteins.

In conclusion, the present study shows that combination of EGFR kinase inhibitors with ionizing radiation could be a potent therapeutic strategy to treat anaplastic oligodendrogliomas characterized by EGFR abnormalities. However, our results suggest that only “EGFR-addictive” tumors highly benefit from this therapeutic combination and, neither molecular makers, such as *EGFR* gene amplification and PTEN loss, nor the basal activation of the EGFR-dependent pathways are reliable markers to select these responsive tumors. In contrast, evaluating the impact of TKI on the EGFR-dependent pathways in few days after treatment onset seems to be more relevant. These promising data require further validation first by using othotopic xenograft models.

## Supporting Information

Figure S1 **Treatment procedures.** (**A**) Treatments started at D1 when tumors reached 250 ± 50 mm^3^ (V_0_) and were administered for two consecutive weeks. Control mice were injected with saline. Gefitinib (GEF) was administered *i.p*. at a daily dose of 75 mg/kg. RT was delivered at a total dose of 20 Gy. In the GEF+RT group, mice received GEF 4 h before RT. **(B) Tumor excision for morphological and biological analysis** Tumors were excised 24 h after the last treatment administration at the end of the first (D6) or the second week (D13).
(PDF)

Figure S2 **Tumor lines characterization for EGFR and PTEN status.** (**A**) CGH array profiles of TCG2, TCG3 and TCG4 xenografts. (**B**) Analysis of EGFR gene amplification based on fluorescent in situ hybridization (FISH) assay on TCG2, TCG3 and TCG4 xenografts. (**C**) PTEN expression analysis by western-blotting for TCG2, TCG3 and TCG4 xenografts.
(PDF)

Figure S3 **Response of subcutaneous glioma xenografts to antitumoral treatments.** (**A**) TCG2 (**B**) TCG3 and (**C**) TCG4 xenografts-bearing mice were randomly assigned into four groups (6–14 mice / group): CTRL (solid black line), GEF (solid grey line), RT (dashed black line) and GEF+RT (dashed grey line). Treatments started at D1. Treatments were administered for two consecutive weeks. Results are expressed as Kaplan-Meier plots, considering the percentage of tumors not having reached 5V0 as the survival endpoint.
(PDF)

Figure S4 **(A) Hematoxylin-eosin-safran stained sections of TCG2 and TCG3 xenografts stained with standard (X 40 and X 200 magnification).** For both models, tumors exhibit high cellular density and high proliferative activity with few atypical mitosis. **(B) Morphological analysis in TCG2 glioma xenografts.** Tumors were harvested on D6, 24 h after the last treatment fraction. Representative micrographs of TCG2 xenogratfs sections after saline (CTRL), GEF, RT or GEF+RT treatment (HES staining). **(C) Effects of treatments on cell proliferation in TCG2 glioma xenografts.** The proliferative index corresponds to the percentage of tumor cells positively labeled for Ki-67.
(PDF)

Figure S5 **Immunohistochemical detection of tumor blood vessels.** Based on (**A**) mouse CD31 staining and (**B**) mouse type IV collagen in TCG3 xenografts when mice received either saline (CTRL), GEF, RT or GEF+RT. Tumors were harvested either on day 6 or day 13. A well superimposition of stainings was noticed in each case.
(PDF)

Figure S6 **Immunohistochemical detection of tumor blood vessels.** (**A**) Based on mouse CD31 staining and type IV collagen in TCG2 xenograft showing a well superimposition of stainings. (**B**) based on type IV collagen staining in TCG2 xenografts showing no vascular change when mice received either saline (CTRL), GEF, RT or GEF+RT.
(PDF)

Methods S1 **Supplementary detailed methods.**
(PDF)

Table S1 **Tumor lines characterization for oncogenic alteration commonly found in high-grade glioma.**
(PDF)
