# Supplementary figures and images for: Synergistic Antitumor Effect between Gefitinib and Fractionated Irradiation in Anaplastic Oligodendrogliomas Cannot Be Predicted by the Egfr Signaling Activity
Source: PLoS One. 2013 Jul 18;8(7):e68333. doi: 10.1371/journal.pone.0068333 (PMC3715478; doi:10.1371/journal.pone.0068333)

**A TREATMENT PROCEDURES**

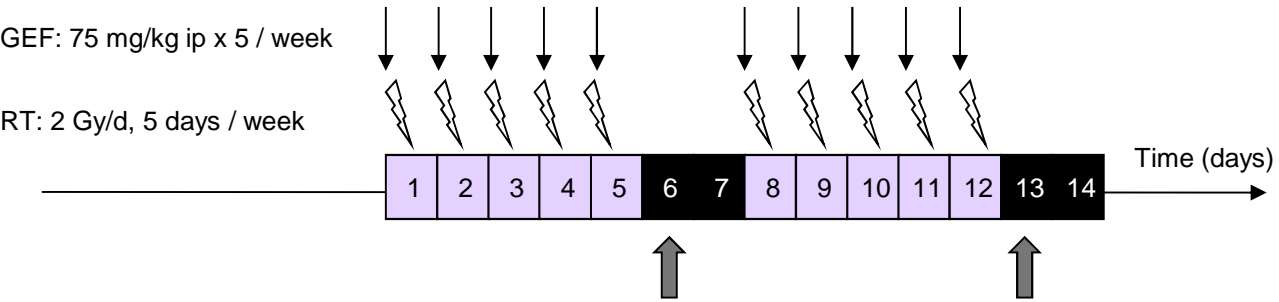

**B TUMOR EXCISIONS FOR MORPHOLOGICAL AND BIOLOGICAL ANALYSIS**

Figure S1

Supplement: Figure S1 — Treatment procedures. (A) Treatments started at D1 when tumors reached 250 ± 50 mm3 (V0) and were administered for two consecutive weeks. Control mice were injected with saline. Gefitinib (GEF) was administered i.p. at a daily dose of 75 mg/kg. RT was delivered at a total dose of 20 Gy. In the GEF+RT group, mice received GEF 4 h before RT. (B) Tumor excision for morphological and biological analysis Tumors were excised 24 h after the last treatment administration at the end of the first (D6) or the second week (D13). (PDF) [file pone.0068333.s001.pdf]

A

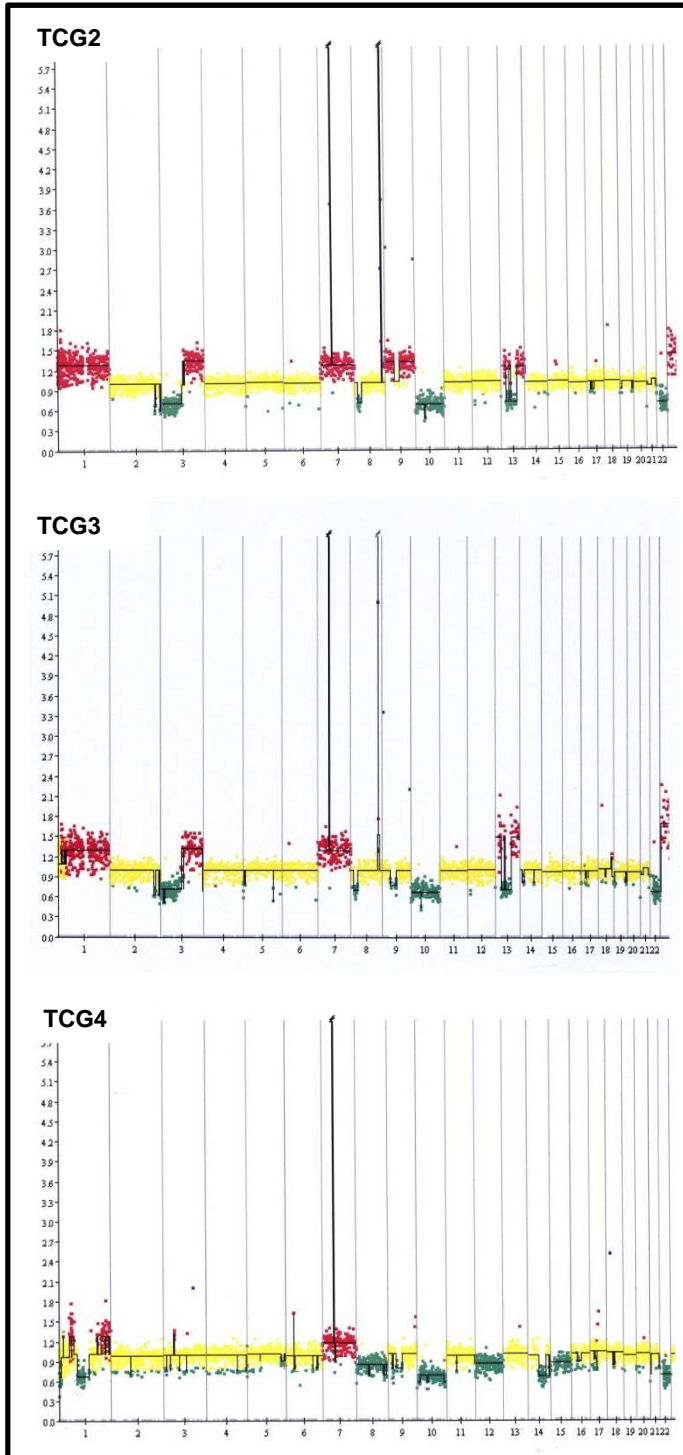

B

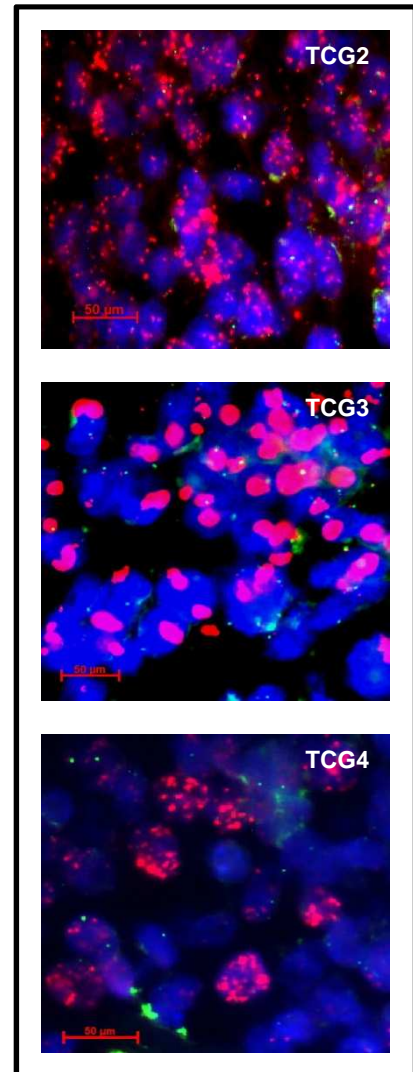

C

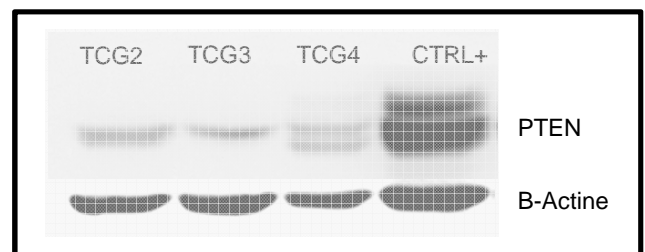

Figure S2

Supplement: Figure S2 — Tumor lines characterization for EGFR and PTEN status. (A) CGH array profiles of TCG2, TCG3 and TCG4 xenografts. (B) Analysis of EGFR gene amplification based on fluorescent in situ hybridization (FISH) assay on TCG2, TCG3 and TCG4 xenografts. (C) PTEN expression analysis by western-blotting for TCG2, TCG3 and TCG4 xenografts. (PDF) [file pone.0068333.s002.pdf]

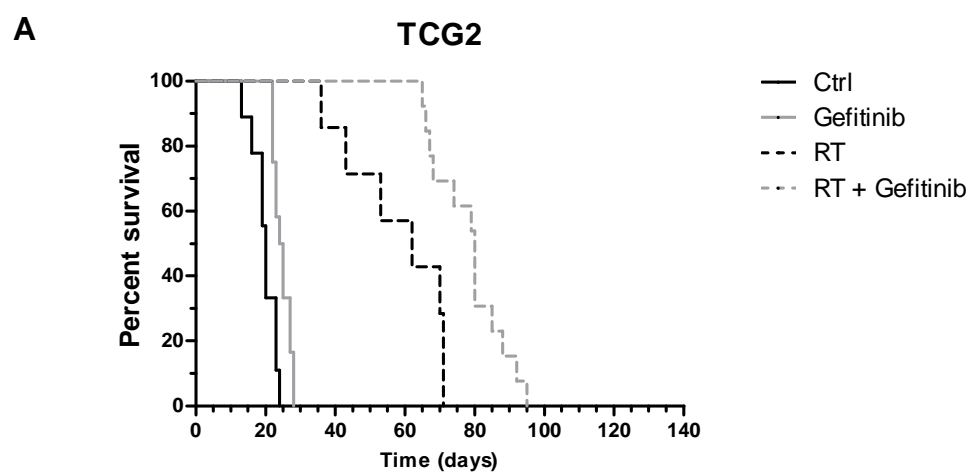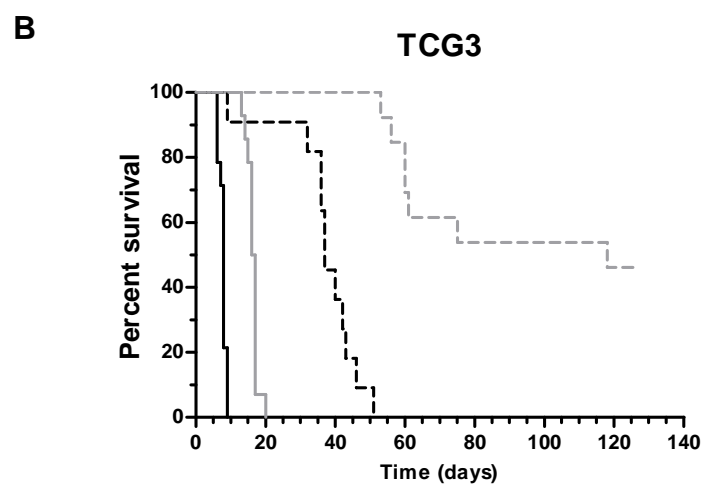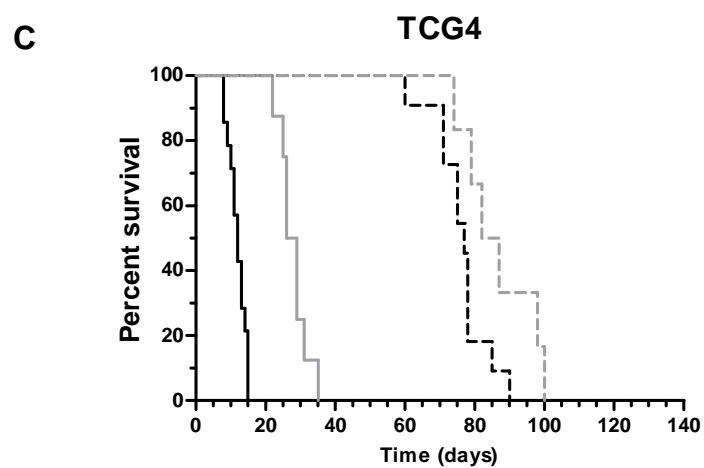

Figure S3

Supplement: Figure S3 — Response of subcutaneous glioma xenografts to antitumoral treatments. (A) TCG2 (B) TCG3 and (C) TCG4 xenografts-bearing mice were randomly assigned into four groups (6–14 mice / group): CTRL (solid black line), GEF (solid grey line), RT (dashed black line) and GEF+RT (dashed grey line). Treatments started at D1. Treatments were administered for two consecutive weeks. Results are expressed as Kaplan-Meier plots, considering the percentage of tumors not having reached 5V0 as the survival endpoint. (PDF) [file pone.0068333.s003.pdf]

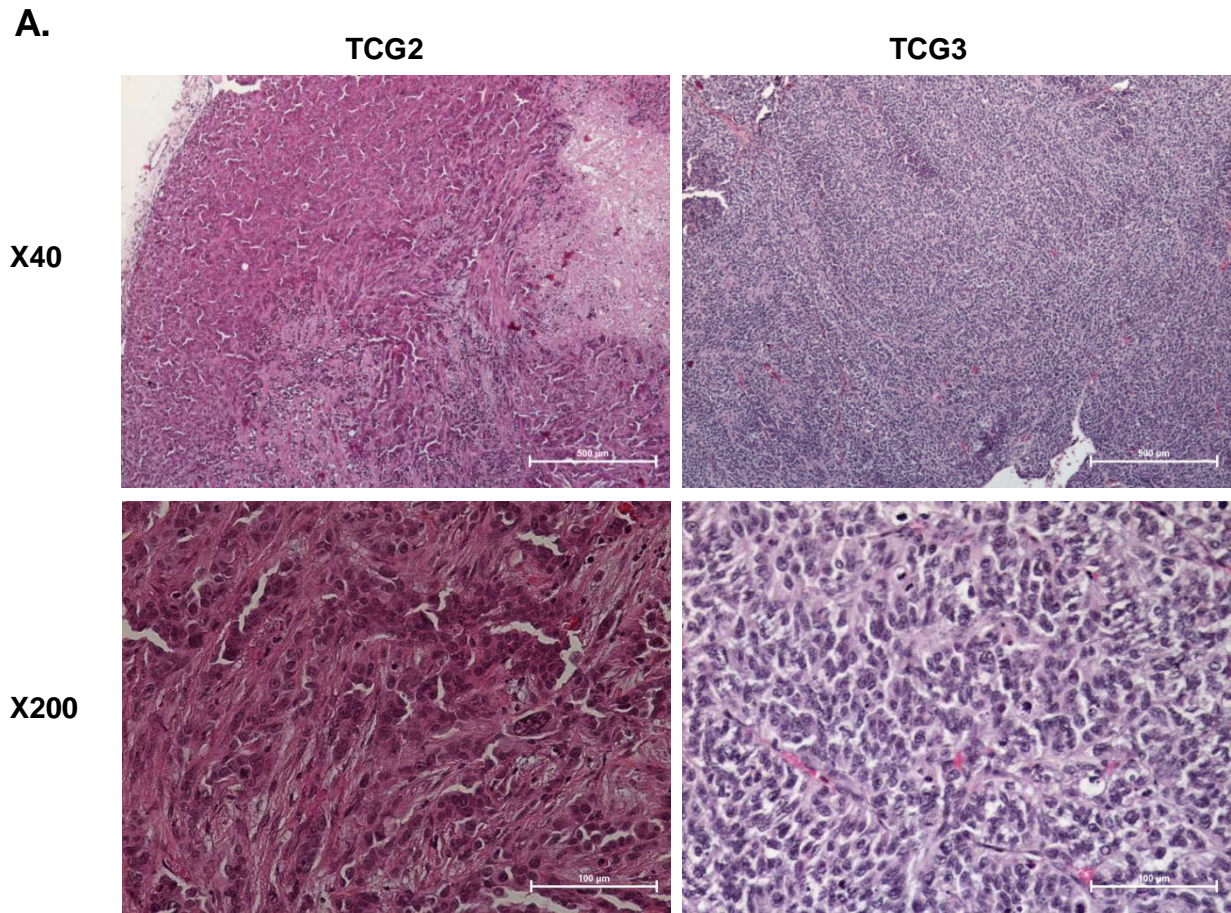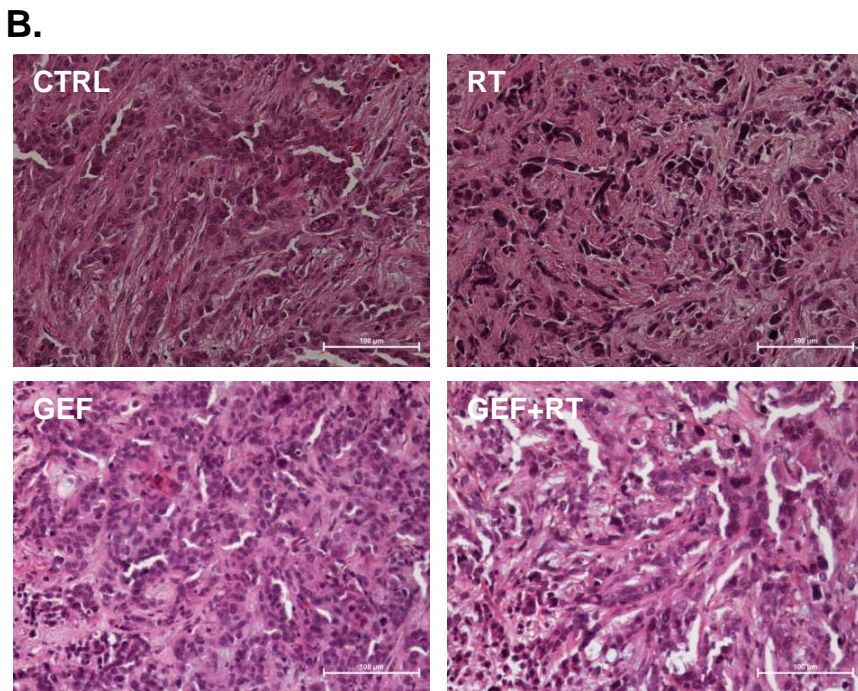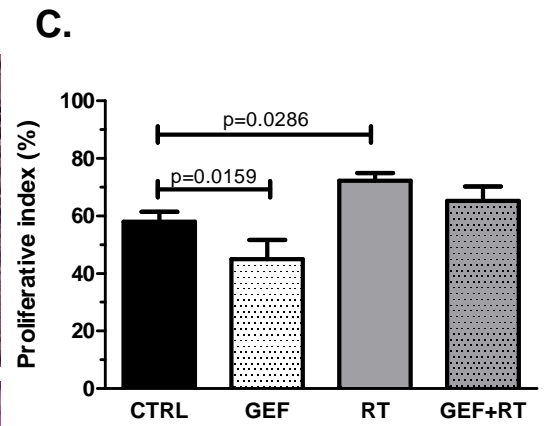

Figure S4

Supplement: Figure S4 — (A) Hematoxylin-eosin-safran stained sections of TCG2 and TCG3 xenografts stained with standard (X 40 and X 200 magnification). For both models, tumors exhibit high cellular density and high proliferative activity with few atypical mitosis. (B) Morphological analysis in TCG2 glioma xenografts. Tumors were harvested on D6, 24 h after the last treatment fraction. Representative micrographs of TCG2 xenogratfs sections after saline (CTRL), GEF, RT or GEF+RT treatment (HES staining). (C) Effects of treatments on cell proliferation in TCG2 glioma xenografts. The proliferative index corresponds to the percentage of tumor cells positively labeled for Ki-67. (PDF) [file pone.0068333.s004.pdf]

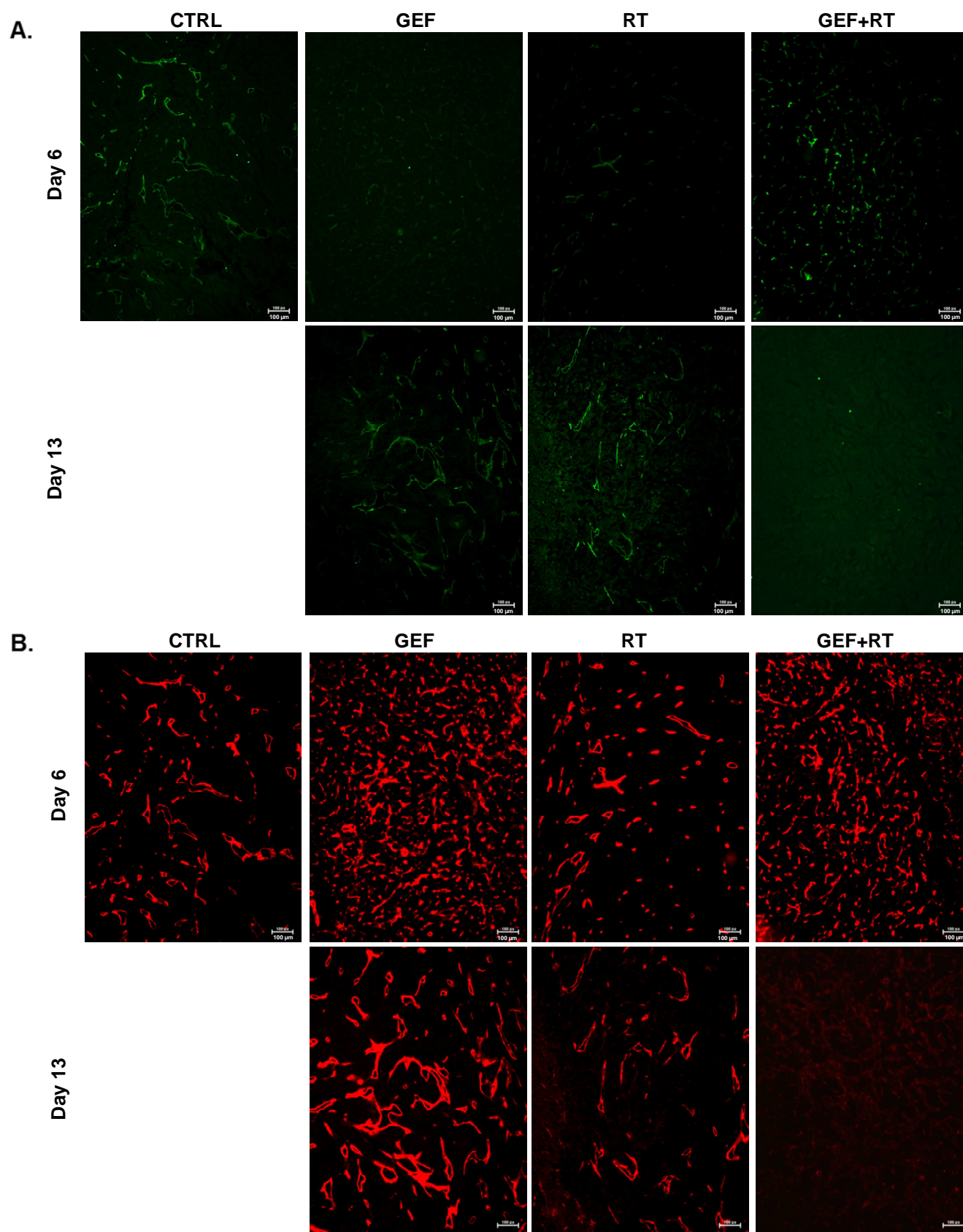

Figure S5

Supplement: Figure S5 — Immunohistochemical detection of tumor blood vessels. Based on (A) mouse CD31 staining and (B) mouse type IV collagen in TCG3 xenografts when mice received either saline (CTRL), GEF, RT or GEF+RT. Tumors were harvested either on day 6 or day 13. A well superimposition of stainings was noticed in each case. (PDF) [file pone.0068333.s005.pdf]

**A.**

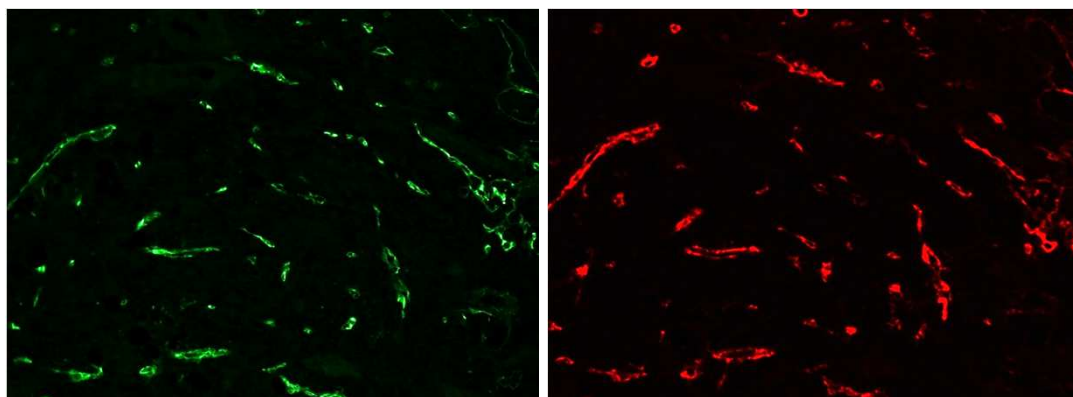

**B.**

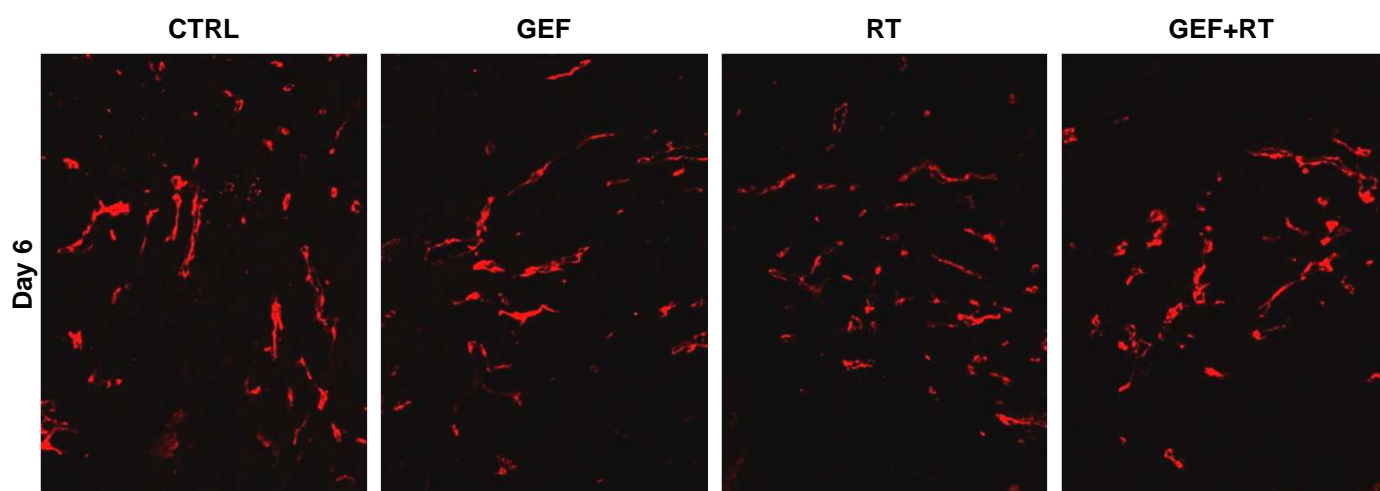

Figure S6

Supplement: Figure S6 — Immunohistochemical detection of tumor blood vessels. (A) Based on mouse CD31 staining and type IV collagen in TCG2 xenograft showing a well superimposition of stainings. (B) based on type IV collagen staining in TCG2 xenografts showing no vascular change when mice received either saline (CTRL), GEF, RT or GEF+RT. (PDF) [file pone.0068333.s006.pdf]
