# Supplementary material for: Synergistic Antitumor Effect between Gefitinib and Fractionated Irradiation in Anaplastic Oligodendrogliomas Cannot Be Predicted by the Egfr Signaling Activity
Source: PLoS One. 2013 Jul 18;8(7):e68333. doi: 10.1371/journal.pone.0068333 (PMC3715478; doi:10.1371/journal.pone.0068333)
Supplement: Methods S1 — Supplementary detailed methods. (PDF) [file pone.0068333.s007.pdf]

METHODS S1

Protein extraction

At the end of the first week of treatment, tumor samples were collected, frozen in liquid nitrogen and kept at -80˚C. Frozen tumor tissue samples (0.3–0.5 g) were homogenized in lysis buffer *(Nuclear extract kit, Active Motif, Belgium)* according to the manufacturer's recommendations (section "Preparation of whole cells extract from tissues"). After centrifugation (10,000 g for 10 min at 4˚C), supernatants were collected and the protein concentration was measured. Equal amounts of protein were assayed for determination of VEGF concentration and BPA assay.

Phospho-EGFR phospho-AKT and phospho-MEK1 expression using the BPA assay

The expression of phospho-EGFR, phospho-AKT and phospho-MEK1 were analyzed using phosphoprotein array (Bio-Plex®, Marnes-la-Coquette, France). This technique is based on multiplex sandwich bead immunoassays. For each tumor sample, 25 µg of protein extracts in triplicate were transferred into 96-well dishes and diluted with 25 µl buffered solution. Fluorescent capture beads coupled to antibodies directed against the phosphoproteins (phospho-EGFR, phospho-AKT and phospho-MEK1) were mixed, and added into each well and incubated overnight. Following incubation, the plates were washed and incubated with biotinylated antibodies fixing each target protein. Streptavidin–phycoerythrin solution was then added. The analysis consisted in a double laser fluorescence detection allowing simultaneous identification of the target protein through the red fluorescence emission signal of the bead and quantification of the target protein through the fluorescence intensity of phycoerythrin. Results were recorded as mean fluorescence intensities and compared to negative controls. Positive controls consisting of standard protein extracts from cell lines were added to each series. All results were normalized

through the different batches of analyses by the same tumor sample. The expression level of each phosphoprotein was given in an arbitrary unit.

Proliferation and apoptotic index determination

Proliferation and apoptosis indexes were determined by immunohistochemistry. Briefly, tumors were excised and immediately fixed in 10% (v/v) buffered formalin pH 7.4 for 72 h and paraffin-embedded. Serial 5 µm thick sections were cut through the center of the xenografts. After section dewaxing, antigen retrieval was carried out with a 2100 Proteogenix Retriever (Proteogenix) following the manufacturer's instructions. Sections were stained either with a species-unspecific rabbit monoclonal antibody (clone SP6, Lab vision Corporation; diluted 1:100) to the cell cycle-associated antigen Ki-67, or with an anti-cleaved caspase-3 rabbit anti-serum (BD Biosciences; diluted 1:1000). Primary antibodies were applied overnight at 4°C. Detection of the tissue-bound primary antibodies was performed using the biotinylated antibody/streptavidin-peroxidase detection system. Bound peroxidase was identified using the Novared™ detection system.

To determine proliferation and apoptotic indexes, the percentage of positively labeled tumor cells for at least 1,000 counted tumor cells was recorded in independent areas of the main tumor masses which were free of necrosis.
