# Supplementary material for: Synergistic Antitumor Effect between Gefitinib and Fractionated Irradiation in Anaplastic Oligodendrogliomas Cannot Be Predicted by the Egfr Signaling Activity
Source: PLoS One. 2013 Jul 18;8(7):e68333. doi: 10.1371/journal.pone.0068333 (PMC3715478; doi:10.1371/journal.pone.0068333)
Supplement: Table S1 — Tumor lines characterization for oncogenic alteration commonly found in high-grade glioma. (PDF) [file pone.0068333.s008.pdf]

Table S1: Tumor lines characterization for oncogenic alterations commonly found in high-grade gliomas

Tumor lines	Initial patient diagnosis[1]	Molecular Characteristics							
		EGFR and EGFR downstream signaling pathway						MGMT promoter methylation[8]	p53 status[9]
		EGFR amplification[2]	EGFR variant III[3]	Phospho-EGFR expression level[4]	PTEN expression[5]	PIK3CA mutations (exon 9 & 20)[6]	BRAF mutation status[7]		
**TCG1**	GBM	**+**	*N.D.*	41	**-**	WT	WT	M	WT
**TCG2**	AO	**+**	**-**	679	**-**	WT	WT	M	MUT
**TCG3**	AO	**+**	**-**	2148	**-**	WT	WT	M	MUT
**TCG4**	AO	**+**	**+**	195	**-**	WT	WT	M	WT
**TCG7**	GBM	**+**	*N.D.*	57	*N.D.*	WT	WT	U	WT
**TCG9**	GBM	*N.D.*	**-**	37	**+**	WT	WT	U	WT
**TCG11**	AO	*N.D.*	**-**	22	**+**	WT	MUT D594G	M	WT
**TCG13**	GBM	*N.D.*	**-**	9	**+**	WT	WT	M	MUT
**TCG15**	GBM	*N.D.*	**-**	11	**+**	WT	WT	M	MUT
**TCG17**	GBM	*N.D.*	*N.D.*	19	**-**	WT	MUT V600E	U	WT

[1] Primary diagnoses (determined in 2001 by a first pathologist according to the WHO classification 2000) were compared to a second opinion (given by an independent pathologist in 2013 according to the WHO classification 2007). AO = anaplastic oligodendroglioma

[2] EGFR amplification was assessed using CGH array and FISH, leading to consistent results.

[3] The expression of the EGFR variant III was determined by western-blotting.

[4] Phospho-EGFR expression level was determined using the Bio-plex phosphotrein arrays

[5] PTEN status was determined after gene expression analysis using qRT-PCR and protein expression analysis by western-blotting: (+) = detectable or (-) = undetectable. *N.D.* = not determined

[6] PIK3CA mutation analysis (exons 9 & 20) was performed using direct sequencing

[7] Mutation in exon 15 of *BRAF* was assessed using LightCycler PCR and fluorescent melting curves analysis and confirmed by direct sequencing: WT = wild type or MUT = Mutated *(Rowe, Cytojournal, 2006)*

[8] MGMT promotor methylation status was evaluated with the methylation specific polymerase chain reaction after DNA modification by sodium bisulfite *(Herman et al, PNAS, 1996)*: M = mutated or U = unmethylated

[9] p53 status was determined by FASAY *(Ishioka, Nature Genetics, 1993)*: WT = wild type or MUT = Mutated
